# Performance assessment on the Korean Computerized Neurobehavioral Test using a mobile device and a conventional computer: an experimental study

**DOI:** 10.1186/s40557-018-0264-6

**Published:** 2018-08-29

**Authors:** Young Seok Byun, Sung Kyu Park, Joon Sakong, Man Joong Jeon

**Affiliations:** 1 Department of Occupational and Environmental Medicine, Yeungnam University Hospital, 3rd floor, Yeong-ui-gwan, 170, Hyeonchung-ro, Namgu, Daegu, 42415 Republic of Korea; 2 Department of Preventive Medicine and Public Health, College of Medicine, Yeungnam University, Yeungnam University Hospital, 170, Hyeonchung-ro, Namgu, Daegu, 42415 Republic of Korea

**Keywords:** Korean computerized neurobehavioral test, Mobile device, Desktop computer, Assessment

## Abstract

**Background:**

The Korean Computerized Neurobehavioral Test (KCNT) is a psychological assessment tool used as part of Workers’ Special Health Examinations in Korea. Due to the spread of mobile technology, this study aimed to compare results of the KCNT administered on a tablet PC versus a desktop computer, and, therefore, assess the clinical applicability of mobile devices.

**Methods:**

A total of 72 participants enrolled in this study. Their age, sex, and years of formal education were collected during an interview, as well as their typing speed. The test battery comprised five subtests: Simple Reaction Time test, Choice Reaction Time test, Digit Addition test, Symbol-Digit Substitution test, and Finger Tapping Speed test. Participants repeated the KCNT test battery in a randomly assigned order using four different testing systems: a desktop computer equipped with a conventional 106-key keyboard (System 1), a desktop computer equipped with a simplified keyboard (System 2), a tablet PC with a simplified 17-key on-screen keyboard (System 3), and a tablet PC equipped with a simplified keyboard (System 4).

**Results:**

Results of the Digit Addition test did not differ significantly for different testing systems. In contrast, results of the Simple Reaction Time test, Choice Reaction Time test, Symbol-Digit Substitution test, and Finger Tapping test were lower for the tablet PC (Systems 3 and 4) compared to the desktop computer (Systems 1 and 2). Systems 1 and 2 did not show significantly different results. Performance on System 3 was inferior to that on System 4, only for the Choice Reaction Time test and Finger Tapping Speed test. There were also significant differences in performance by computer familiarity when adjusted for age and education; however, the performance of each group on the test systems showed similar patterns.

**Conclusions:**

It is not recommended to use a tablet PC to administer the KCNT to evaluate neurobehavioral performance for the Simple Reaction Time test and Choice Reaction Time test; however, tablet PCs with an on-screen keyboard may be used to perform the Digit Addition test, and the Symbol-Digit Substitution test and Finger Tapping Speed test to a limited degree.

## Background

Within recent years, the use of mobile devices such as smartphones and tablet PCs has been growing rapidly. In countries with developing economies in 2013, the rate (median) of adult smartphone users was reported as 21%, and increased to 28 and 37% in 2014 and 2015, respectively [1]. In 2015, adult smartphone ownership was reported to be as high as 68% among economically advanced countries [1]. The number of tablet PC users worldwide also increased from 0.70 billion in 2013 to 0.91 billion in 2014, and was predicted to surpass one billion by 2017, with growth predicted to stay over 10% [2].

Therefore, application of mobile technology in the medical sector has drawn much attention. The World Health Organization addressed the “unprecedented spread of mobile technologies” as a new horizon for health and defined the application of such powerful innovations as “mHealth” in 2011 [3]. In the same year, the Korea Food and Drug Administration (KFDA) also released guidelines for the review and approval of mobile picture archiving and communication systems (PACS) for secure and controlled clinical use [4]. Use of mHealth in clinical settings can already be seen, such as diagnoses using mobile image interpretation of computed tomography (CT) examinations and hospital inpatient rounding programs [5–8].

Based on the above, it is evident that the Korean Computerized Neurobehavioral Test (KCNT) could be applied clinically using mobile devices. The KCNT is a powerful, standardized tool in the assessment of neurobehavioral functions with high sensitivity, fidelity, and validity. It is also a more practical tool compared to interview-based tests such as the WHO Neurobehavioral Core Test Battery (WHO-NCTB) and psychological assessment tools applied as part of the Workers’ Special Health Examinations to screen workers at risk for exposure to neurotoxic chemicals [9–13].

Currently, while performing the KCNT, desktop computers are recommended and preferred over laptop computers, despite desktop computers’ inferior portability. This is because the performance of examinees with lower computer profiency is known to be influenced by the type of computer [14, 15]. In this study, we aimed to primarily evaluate the results of the KCNT performed on a tablet PC versus a desktop computer, and, therefore, assess the clinical applicability of mobile devices.

## Methods

### Participants

This study was conducted from May to December 2017. Participants were selected using convenience sampling. Those who visited the hospital for a health examination were asked to participate and were interviewed for eligibility according to the inclusion and exclusion criteria. Then, they were asked to complete the KCNT. To control confounding variables such as age, sex, and education, every participant repeated the battery of the KCNT using four different test devices (later referred to as *Systems*) in a randomly assigned order. This study was approved by the institutional review board of Yeungnam University (IRB File No. YU 2017-04-001-001). Seventy-four people volunteered and none were ineligible to participate. However, data from two participants were inappropriate for analysis and were excluded. Therefore, 72 participants were included in this study.

### Inclusion and exclusion criteria

People who were aged over 19 and under 65 years were selected if they did not meet the exclusion criteria. They were excluded if they had any of the following [16–20]: a past medical history of or present serious condition that could affect neurobehavioral performance such as head trauma or neurological disease; potential occupational exposure to neurotoxins revealed during an interview; and physical disabilities that could influence the neurobehavioral test, such as hearing impairment, color vision deficiency, or severe lower back pain.

### Interview

General characteristics were collected during the interview, including age, sex, and years of formal education. Participants’ typing speed was also tested to objectively evaluate computer familiarity. Typing speed was defined as the number of Korean characters typed in a minute.

### Testing systems

**Fig. 1:**
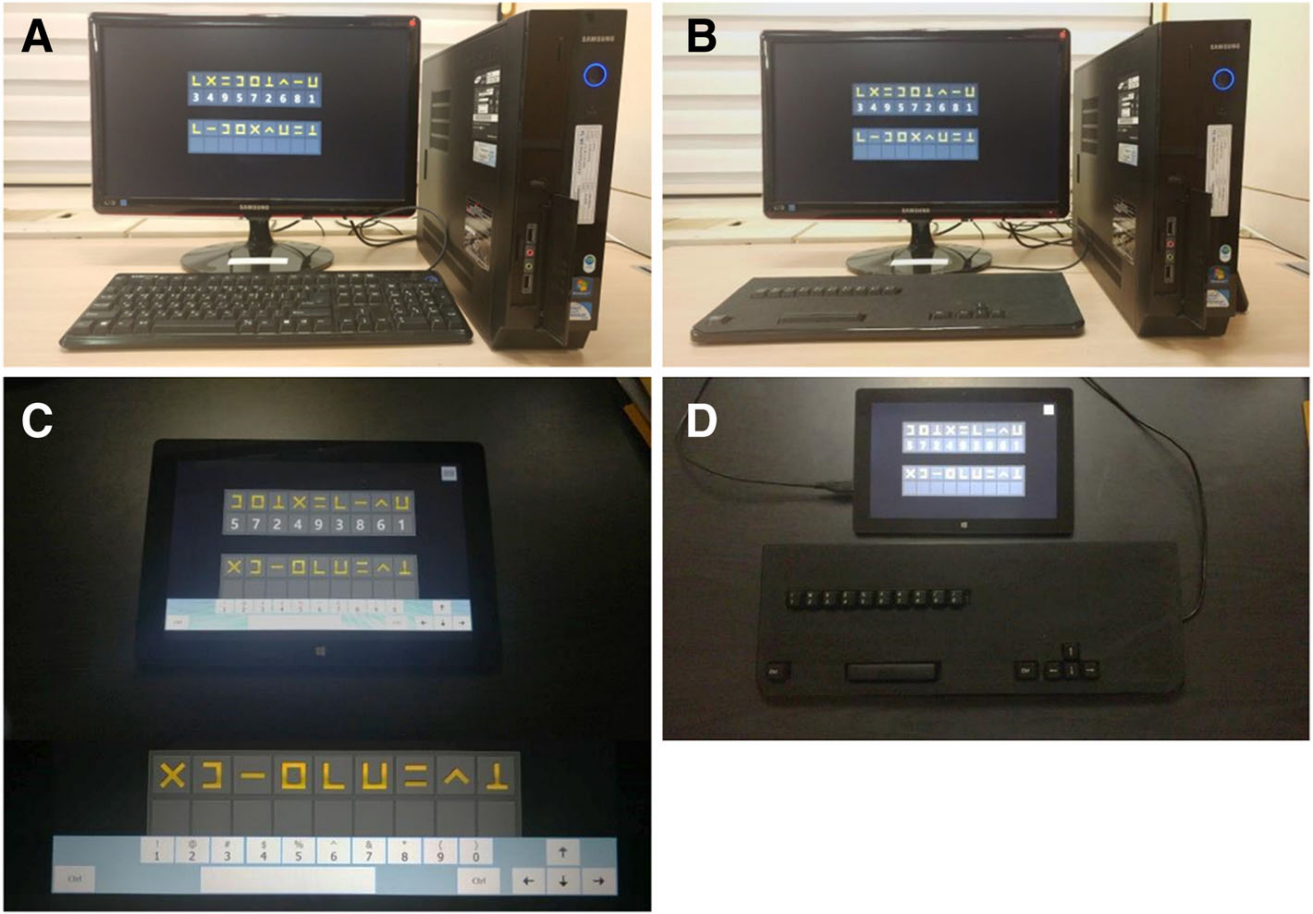
The test systems. Four different test systems were used in this study: **a** System 1, a desktop computer equipped with a conventional 106-key keyboard; **b** System 2, a desktop computer equipped with a simplified 17-key keyboard; **c** System 3, a tablet PC with an on-screen keyboard; **d** System 4, a tablet PC equipped with a simplified 17-key keyboard

Four different testing systems were used in this study (Fig. 1): a desktop computer equipped with a conventional 106-key keyboard (System 1), a desktop computer equipped with a simplified keyboard (System 2), a tablet PC with a simplified 17-key on-screen keyboard (System 3), and a tablet PC equipped with a simplified keyboard (System 4). In System 3, the tablet PC display was a capacitive screen digitizer and an on-screen keyboard was used as the input device. The tablet PC used in this study had a display with a diagonal length of 10 in., whereas the monitor connected to the desktop computer had a display with a diagonal length of 24 in..

### Korean computerized neurobehavioral test

The test battery comprised five subtests selected by the authors [9, 14, 15]: Simple Reaction Time (SRT) test, Choice Reaction Time (CRT) test, Digit Addition (DA) test, Symbol-Digit Substitution (SDS) test, and Finger Tapping Speed (FTS) test. Since every participant performed the KCNT multiple times, there was a risk of biases due to mental fatigue and learning effect [21, 22]. To minimize these biases, participants performed the test in a completely counter-balanced, randomly and evenly assigned order. That is, there were 24 possible combinations of the four systems, and every participant was assigned a random sequence in which to serially perform the KCNT.

### Parameters

All subtests, except for the FTS test, had three common parameters: correct response rate (Rate_CR_), mean reaction time of correct responses (RT_mean_), and standard deviation of the reaction time (SD_RT_), where reaction time is expressed in milliseconds. In contrast, there were only two parameters for the FTS test: average number of taps during 10-s trials using the dominant hand and the non-dominant hand, respectively (FTS_D_ and FTS_ND_).

### Statistical analysis

Statistical analyses were performed using IBM SPSS Statistics Version 22. General characteristics were described using frequencies, percentages, means, and standard deviations. To assess performance, parameters generated from each subtest were analyzed. Repeated measures analysis of variance (ANOVA) and the Friedman test were used to compare performance for the four different systems. For the ANOVA and Friedman test, Bonferroni test and Wilcoxon signed-rank test were applied as post-hoc procedures, respectively. Analysis of covariance (ANCOVA) was used to compare performance between groups with different computer familiarity. For ANCOVA, Bonferroni test was applied as a post-hoc procedure. A *p*-value below 0.05 was considered statistically significant.

## Results

### General characteristics

General characteristics of all 72 participants are listed and summarized in Table 1. Participants were on average 40.3 ± 12.8 years old and 50% were male. Seventy-one participants (98.6%) completed high school education or higher; one participant completed only middle school education. Their typing speed was 258.2 ± 164.5 characters per minute on average. Forty participants (55.6%) could type more than 200 characters per minute, and were classified as Group III (very familiar with computers). Twenty-three participants (31.9%) had a typing speed of lower than 200 characters per minute and were classified as Group II (relatively familiar with computers). Nine participants (12.5%) with a typing speed of near zero were classified as Group I (no competency using computers).

**Table 1:** General characteristics of all participants

Characteristics	n (%)	Mean (SD)
Age (years)		
20–29	22 (30.6)	40.3 (12.8)
30–39	14 (19.4)	
40–49	14 (19.4)	
50–59	16 (22.2)	
≥ 60	6 (8.4)	
Sex		
Male	36 (50.0)	
Female	36 (50.0)	
Education		
Elementary school	0 (0.0)	
Middle school	1 (1.4)	
High school	14 (19.5)	
University/College	52 (72.2)	
Post-graduate school	5 (6.9)	
Typing speed^a^		
Group I	9 (12.5)	258.2 (164.5)
Group II	23 (31.9)	
Group III	40 (55.6)	
Total	72 (100.0)	

*SD* standard deviation, *Group I* participants with typing speed of near zero (no competency using computers), *Group II* participants with typing speed less than 200 characters/min (relatively familiar with computers), *Group III* participants with typing speed of 200 characters/min or greater (very familiar with computers)

^a^Number of Korean characters typed in a minute

### Performance on the KCNT by test system

Performance of all participants was evaluated by comparing test parameters among Systems 1, 2, 3, and 4 (Table 2). The mean reaction time of the SRT test showed a significant difference between systems (*p* < 0.001). Post-hoc analysis showed that the mean reaction time for Systems 3 and 4 was greater than that of Systems 1 and 2. The mean reaction time for the CRT test also showed differences between systems (*p* < 0.001). In contrast to the results from the SRT test, post-hoc analysis showed that the mean reaction time for the CRT test using System 3 was significantly greater than that of System 4. The mean reaction time for the CRT test using Systems 3 and 4 was significantly greater than that of Systems 1 and 2. Although the mean reaction time for the SDS test was similar between systems (*p* = 0.961), the correct response rate significantly differed (*p* < 0.001); there was no difference between System 1 and System 2, but the rate was lower for System 3. Performance on the DA test did not show significant differences by system type for all parameters (RT_mean_, *p* = 0.364; SD_RT_, *p* = 0.664; Rate_CR_, *p* = 0.751). Similarly, for the FTS test, results using dominant hand did not differ between systems (*p* = 0.350), but the results using non-dominant hand showed a decreased performance in System 3 compared to Systems 1, 2 and 4 (*p* < 0.001).

**Table 2:** Performance on the KCNT between test systems

KCNT	Type of system				*F*-value	*p*-value^*^	Post-hoc^a^
	System 1	System 2	System 3	System 4			
	Mean (SD)	Mean (SD)	Mean (SD)	Mean (SD)			
SRT							
RT_mean_	391.0 (133.6)	381.1 (131.6)	532.7 (177.7)	510.3 (193.4)	150.670	< 0.001	(3 = 4) > (1 = 2)
SD_RT_	71.5 (70.3)	72.2 (47.0)	115.0 (133.9)	123.1 (270.1)	2.528	0.098	
Rate_CR_	0.998 (0.010)	1.000 (0.004)	0.997 (0.010)	0.997 (0.011)	1.282	0.282	
CRT							
RT_mean_	590.6 (107.4)	593.0 (117.2)	750.4 (116.1)	691.9 (109.2)	171.785	< 0.001	3 > 4 > (2 = 1)
SD_RT_	95.2 (35.6)	105.8 (53.6)	124.8 (58.0)	114.8 (47.2)	6.105	0.001	(3 = 4) > 1
Rate_CR_	0.994 (0.014)	0.994 (0.013)	0.991 (0.018)	0.996 (0.009)	1.714	0.176	
DA							
RT_mean_	2518.4 (534.1)	2498.6 (496.7)	2557.8 (463.4)	2513.2 (545.6)	1.068	0.364	
SD_RT_	497.3 (320.6)	508.9 (351.1)	539.9 (287.2)	527.8 (375.8)	0.528	0.664	
Rate_CR_	0.865 (0.161)	0.852 (0.144)	0.870 (0.148)	0.862 (0.161)	0.403	0.751	
SDS							
RT_mean_	2016.7 (397.8)	2018.4 (339.2)	2013.3 (385.3)	2025.9 (364.8)	0.099	0.961	
SD_RT_	534.4 (369.6)	534.1 (200.7)	616.9 (286.2)	612.1 (255.0)	2.624	0.058	
Rate_CR_	0.988 (0.023)	0.988 (0.020)	0.970 (0.042)	0.978 (0.036)	6.824	< 0.001	3 < (2 = 1)
FTS							
FTS_D_	72.0 (8.5)	72.3 (8.3)	71.3 (9.5)	72.0 (8.3)	1.053	0.350	
FTS_ND_	65.4 (8.9)	66.2 (9.2)	63.0 (9.4)	66.2 (9.0)	13.378	< 0.001	(2 = 4 = 1) > 3

*System 1* a desktop computer equipped with a conventional 106-key keyboard, *System 2* a desktop computer equipped with a simplified 17-key keyboard, *System 3* a tablet PC with an on-screen keyboard, *System 4* a tablet PC equipped with a simplified 17-key keyboard, *SD* standard deviation, *SRT* simple reaction time, *CRT* choice reaction time, *DA* digit addition, *SDS* symbol-digit substitution, *FTS* finger tapping speed, *RT*_*mean*_ mean reaction time, *SD*_*RT*_ standard deviation of reaction time, *Rate*_*CR*_ correct response rate, *FTS*_*D*_ finger tapping speed of dominant hand, *FTS*_*ND*_ finger tapping speed of non-dominant hand

RT_mean_ and SD_RT_ are in millisecond (ms); FTS_D_ and FTS_ND_ are in average number of taps per 10 s

^*^Calculated by repeated measures ANOVA

^a^The numbers 1, 2, 3, and 4 represent Systems 1, 2, 3, and 4, respectively. Analyzed by Bonferroni

### Performance on the KCNT between computer familiarity groups by test system

If performance differed by computer familiarity group, further analysis would be required to compare test systems stratifying by computer familiarity. Therefore, test results from Groups I, II, and III were compared for different systems (Table 3), even though this study did not primarily aim to assess the effect of computer familiarity on the performance of computerized neurobehavioral tests. Since the general characteristics differed by each group, the results had to be adjusted by age and the length of formal education.

**Table 3:** Performance on the KCNT between computer familiarity groups by test systems

KCNT		Type of system	Computer familiarity			*F*-value	*p*-value^†^	Post-hoc^b^
			Group I	Group II	Group III			
			Mean (SE)^a^	Mean (SE)^a^	Mean (SE)^a^			
SRT	RT_mean_	System 1	265.3 (58.1)	422.7 (30.6)	401.0 (26.9)	4.353	0.017	2 > 1
		System 2	222.4 (56.6)	407.7 (29.8)	401.6 (26.2)	6.126	0.004	2 > 1
		System 3	341.4 (72.7)	568.0 (38.3)	555.4 (33.7)	5.576	0.006	2 > 1
		System 4	333.2 (83.3)	540.1 (43.9)	533.0 (38.6)	3.521	0.035	2 > 1
	SD_RT_	System 1	49.1 (35.0)	65.6 (18.4)	79.9 (16.2)	0.231	0.794	
		System 2	46.7 (22.9)	78.8 (12.1)	74.2 (10.6)	1.169	0.317	
		System 3	51.0 (65.5)	98.5 (34.5)	138.8 (30.3)	0.537	0.587	
		System 4	64.7 (130.5)	128.6 (68.8)	133.1 (60.4)	0.135	0.874	
	Rate_CR_	System 1	0.994 (0.005)	0.997 (0.002)	0.999 (0.002)	0.303	0.739	
		System 2	1.000 (0.002)	1.000 (0.001)	1.000 (0.001)	0.032	0.969	
		System 3	0.993 (0.005)	0.997 (0.003)	0.999 (0.002)	0.435	0.649	
		System 4	0.991 (0.005)	0.999 (0.003)	0.997 (0.002)	1.596	0.210	
CRT	RT_mean_	System 1	616.5 (39.4)	592.5 (20.8)	583.7 (18.2)	0.236	0.791	
		System 2	645.6 (48.1)	594.5 (25.3)	580.3 (22.3)	0.679	0.511	
		System 3	735.0 (42.0)	745.2 (22.1)	756.9 (19.4)	0.085	0.919	
		System 4	721.1 (41.0)	679.3 (21.6)	692.6 (19.0)	0.697	0.502	
	SD_RT_	System 1	119.7 (16.1)	112.9 (8.5)	79.5 (7.5)	3.160	0.049	2 > 3
		System 2	124.5 (25.4)	127.6 (13.4)	89.1 (11.8)	1.698	0.191	
		System 3	116.6 (28.5)	135.7 (15.0)	120.4 (13.2)	0.500	0.609	
		System 4	101.0 (22.6)	120.6 (11.9)	114.7 (10.5)	0.492	0.614	
	Rate_CR_	System 1	0.985 (0.007)	0.997 (0.004)	0.994 (0.003)	1.920	0.155	
		System 2	0.995 (0.006)	0.998 (0.003)	0.992 (0.003)	0.900	0.411	
		System 3	0.995 (0.009)	0.988 (0.005)	0.992 (0.004)	0.403	0.670	
		System 4	0.987 (0.004)	0.995 (0.002)	0.999 (0.002)	2.119	0.128	
DA	RT_mean_	System 1	2684.4 (248.5)	2803.4 (131.0)	2317.1 (115.1)	3.006	0.056	
		System 2	2768.9 (238.0)	2660.4 (125.5)	2344.8 (110.3)	1.349	0.267	
		System 3	2644.9 (217.0)	2727.1 (114.4)	2440.9 (100.5)	1.394	0.255	
		System 4	2617.7 (265.7)	2703.8 (140.1)	2380.0 (123.1)	1.178	0.314	
	SD_RT_	System 1	466.1 (157.4)	593.9 (83.0)	448.7 (72.9)	1.059	0.353	
		System 2	565.0 (174.6)	500.2 (92.1)	501.3 (80.9)	0.079	0.925	
		System 3	641.2 (141.3)	628.9 (74.5)	465.9 (65.5)	0.966	0.386	
		System 4	696.8 (184.7)	638.1 (97.4)	426.3 (85.6)	0.986	0.379	
	Rate_CR_	System 1	0.645 (0.067)	0.853 (0.036)	0.921 (0.031)	5.830	0.005	1 < (2 = 3)
		System 2	0.625 (0.059)	0.791 (0.031)	0.939 (0.027)	8.408	0.001	1 < 2 < 3
		System 3	0.680 (0.061)	0.868 (0.032)	0.913 (0.028)	5.544	0.006	1 < (2 = 3)
		System 4	0.704 (0.070)	0.857 (0.037)	0.901 (0.033)	2.852	0.065	
SDS	RT_mean_	System 1	2328.7 (143.1)	2045.3 (75.4)	1930.0 (66.3)	2.564	0.085	
		System 2	2280.2 (111.0)	2006.8 (58.5)	1966.2 (51.4)	3.461	0.037	1 > 2
		System 3	2306.9 (120.9)	1984.4 (63.7)	1963.8 (56.0)	4.019	0.022	1 > 2
		System 4	2215.2 (129.2)	2021.8 (68.1)	1985.7 (59.8)	1.292	0.282	
	SD_RT_	System 1	836.6 (164.4)	521.8 (86.7)	473.7 (76.2)	2.095	0.131	
		System 2	635.8 (94.5)	460.4 (49.8)	553.6 (43.8)	2.822	0.067	
		System 3	819.3 (135.8)	585.8 (71.6)	589.2 (62.9)	1.681	0.194	
		System 4	709.1 (118.7)	558.1 (62.6)	621.3 (55.0)	1.183	0.313	
	Rate_CR_	System 1	0.979 (0.011)	0.989 (0.006)	0.990 (0.005)	0.466	0.630	
		System 2	0.991 (0.010)	0.993 (0.005)	0.984 (0.005)	0.656	0.522	
		System 3	0.946 (0.021)	0.968 (0.011)	0.976 (0.010)	0.740	0.481	
		System 4	0.962 (0.018)	0.982 (0.009)	0.979 (0.008)	0.791	0.458	
FTS	FTS_D_	System 1	69.6 (3.9)	69.7 (2.0)	73.8 (1.8)	0.820	0.445	
		System 2	69.3 (3.7)	70.0 (2.0)	74.2 (1.7)	0.913	0.406	
		System 3	69.1 (4.3)	71.6 (2.3)	71.5 (2.0)	0.186	0.830	
		System 4	68.6 (3.9)	70.9 (2.1)	73.4 (1.8)	0.457	0.635	
	FTS_ND_	System 1	59.2 (4.0)	64.5 (2.1)	67.2 (1.8)	1.273	0.287	
		System 2	58.2 (4.1)	65.1 (2.2)	68.7 (1.9)	2.058	0.136	
		System 3	57.0 (4.2)	63.2 (2.2)	64.3 (1.9)	1.279	0.285	
		System 4	59.1 (4.1)	67.4 (2.2)	67.2 (1.9)	2.327	0.105	

*System 1* a desktop computer equipped with a conventional 106-key keyboard, *System 2* a desktop computer equipped with a simplified 17-key keyboard, *System 3* a tablet PC with an on-screen keyboard, *System 4* a tablet PC equipped with a simplified 17-key keyboard, *SE* standard error, *SRT* simple reaction time, *CRT* choice reaction time, *DA* digit addition, *SDS* symbol-digit substitution, *FTS* finger tapping speed, *RT*_*mean*_ mean reaction time, *SD*_*RT*_ standard deviation of reaction time, *Rate*_*CR*_ correct response rate, *FTS*_*D*_ finger tapping speed of dominant hand, *FTS*_*ND*_ finger tapping speed of non-dominant hand, *Group I* participants with typing speed of near zero (no competency using computers), *Group II* participants with typing speed less than 200 characters/min (relatively familiar with computers), *Group III* participants with typing speed of 200 characters/min or greater (very familiar with computers)

RT_mean_ and SD_RT_ are in millisecond (ms); FTS_D_ and FTS_ND_ are in average number of taps per 10 s

^†^Calculated by ANCOVA

^a^Mean and SE are estimates adjusted by age and education as covariates

^b^The numbers 1, 2, and 3 represent Group I, Group II, and Group III, respectively. Analyzed by Bonferroni

The mean reaction time for the SRT test, correct response rate for the DA test, and mean reaction time for the SDS test showed significant differences among the computer familiarity groups. Post-hoc analyses revealed that the performance of Group II for the SRT test was consistently lower than that of Group I throughout the test systems. Group I had the lowest correct response rate for the DA test when tested with Systems 1, 2, and 3. For the SDS test, Group I showed lower performance in terms of reaction time than Group II when tested with System 2 and 3.

### Performance of KCNT between test systems by computer familiarity group

Since performance differed by computer familiarity group (Table 3), the performance of each group classified by computer familiarity was evaluated by comparing test parameters among Systems 1, 2, 3, and 4 (Table 4).

**Table 4:** Performance on the KCNT between test systems by computer familiarity groups

KCNT		Computer familiarity	Type of system				*F* or χ^2^	*p*-value^*^	Post-hoc^a^
			System 1	System 2	System 3	System 4			
			Mean (SD)	Mean (SD)	Mean (SD)	Mean (SD)			
SRT	RT_mean_	Group I	355.2 (36.8)	320.9 (48.9)	490.2 (28.6)	453.2 (48.2)	60.757	< 0.001	(3 = 4) > 1 > 2
		Group II	425.6 (163.5)	415.3 (148.7)	575.1 (203.2)	535.2 (186.2)	59.296	< 0.001	3 > 4 > (1 = 2)
		Group III	379.1 (126.4)	375.0 (130.3)	517.9 (179.8)	508.9 (216.7)	87.240	< 0.001	(3 = 4) > (1 = 2)
	SD_RT_	Group I	65.6 (41.0)	59.2 (37.1)	92.5 (46.0)	90.8 (40.1)	11.800	0.008	4 > 1
		Group II	70.8 (37.7)	79.8 (51.7)	103.5 (70.8)	101.4 (96.3)	15.887	0.001	3 > (2 = 1)
		Group III	73.2 (88.6)	70.8 (46.5)	126.6 (170.4)	142.8 (355.4)	16.620	0.001	3 > (1 = 2)
	Rate_CR_	Group I	0.997 (0.010)	1.000 (0.000)	0.993 (0.014)	0.993 (0.014)	3.000	0.392	
		Group II	0.999 (0.007)	1.000 (0.000)	0.997 (0.013)	1.000 (0.000)	2.000	0.572	
		Group III	0.998 (0.011)	0.999 (0.005)	0.998 (0.007)	0.996 (0.013)	1.979	0.577	
CRT	RT_mean_	Group I	716.2 (111.3)	714.5 (165.7)	862.8 (73.4)	822.3 (121.2)	15.838	0.001	(3 = 4) > (1 = 2)
		Group II	633.3 (99.3)	630.0 (104.6)	803.0 (92.0)	725.0 (96.1)	51.470	< 0.001	3 > 4 > (1 = 2)
		Group III	537.8 (73.1)	544.3 (81.2)	694.9 (104.6)	643.6 (80.7)	133.278	< 0.001	3 > 4 > (2 = 1)
	SD_RT_	Group I	105.1 (50.6)	100.6 (37.1)	114.1 (21.9)	113.9 (51.9)	0.306	0.820	
		Group II	111.0 (34.5)	123.6 (77.9)	137.9 (70.2)	129.4 (50.0)	2.374	0.499	
		Group III	83.8 (28.5)	96.8 (35.4)	119.7 (55.7)	106.7 (43.6)	22.110	< 0.001	(3 = 4) > 1
	Rate_CR_	Group I	0.993 (0.015)	0.995 (0.009)	0.991 (0.015)	0.993 (0.010)	0.643	0.887	
		Group II	1.000 (0.000)	0.998 (0.006)	0.987 (0.021)	0.997 (0.007)	14.211	0.003	3 < 1
		Group III	0.991 (0.017)	0.991 (0.016)	0.993 (0.017)	0.996 (0.010)	3.182	0.364	
DA	RT_mean_	Group I	2624.4 (534.6)	2709.1 (490.3)	2715.3 (246.9)	2562.7 (270.8)	0.597	0.623	
		Group II	2718.3 (553.1)	2611.4 (525.7)	2738.1 (480.4)	2660.8 (510.7)	5.087	0.166	
		Group III	2379.6 (492.1)	2386.4 (461.8)	2418.8 (451.2)	2417.1 (597.4)	3.540	0.316	
	SD_RT_	Group I	396.0 (230.1)	507.1 (334.2)	530.3 (326.1)	551.9 (413.2)	0.867	0.833	
		Group II	559.9 (384.4)	477.0 (213.9)	580.2 (285.7)	566.5 (319.2)	3.783	0.286	
		Group III	484.0 (296.7)	527.7 (418.0)	518.9 (284.3)	500.1 (403.5)	2.190	0.534	
	Rate_CR_	Group I	0.653 (0.295)	0.688 (0.143)	0.674 (0.298)	0.674 (0.301)	0.067	0.977	
		Group II	0.848 (0.117)	0.810 (0.127)	0.886 (0.097)	0.848 (0.133)	7.415	0.060	
		Group III	0.922 (0.089)	0.914 (0.115)	0.905 (0.079)	0.913 (0.088)	3.375	0.337	
SDS	RT_mean_	Group I	2591.9 (550.5)	2555.1 (307.8)	2647.1 (392.5)	2508.6 (364.8)	1.267	0.737	
		Group II	2132.5 (245.0)	2114.5 (220.6)	2112.9 (283.1)	2157.3 (293.3)	2.113	0.549	
		Group III	1820.6 (261.6)	1842.4 (242.8)	1813.3 (232.1)	1841.7 (260.1)	0.810	0.847	
	SD_RT_	Group I	885.4 (932.6)	691.0 (223.0)	846.0 (362.8)	802.4 (299.2)	3.267	0.352	
		Group II	481.8 (133.3)	491.7 (136.5)	587.7 (146.2)	616.7 (275.4)	4.604	0.014	3 > 1
		Group III	485.7 (166.8)	523.1 (213.9)	582.1 (310.2)	566.6 (216.3)	4.950	0.175	
	Rate_CR_	Group I	0.985 (0.024)	0.988 (0.020)	0.957 (0.028)	0.960 (0.065)	9.182	0.027	^b^
		Group II	0.993 (0.024)	0.993 (0.015)	0.975 (0.041)	0.982 (0.033)	6.843	0.077	
		Group III	0.987 (0.023)	0.985 (0.023)	0.969 (0.045)	0.980 (0.028)	4.727	0.193	
FTS	FTS_D_	Group I	67.7 (10.1)	66.9 (8.4)	65.6 (7.8)	66.9 (9.3)	0.982	0.373	
		Group II	68.7 (6.3)	68.9 (6.3)	69.0 (9.1)	70.0 (6.7)	0.704	0.474	
		Group III	74.9 (8.2)	75.4 (8.1)	73.8 (9.5)	74.3 (8.3)	1.216	0.302	
	FTS_ND_	Group I	57.6 (7.2)	57.6 (7.4)	55.2 (10.2)	57.2 (8.4)	1.418	0.262	
		Group II	63.1 (7.0)	64.4 (7.1)	61.2 (8.4)	66.2 (7.4)	14.175	< 0.001	3 < (2 = 4) and (3 = 1) < 4
		Group III	68.4 (8.9)	69.2 (9.2)	65.9 (8.7)	68.2 (9.0)	18.223	< 0.001	3 < (1 = 2)

*System 1* a desktop computer equipped with a conventional 106-key keyboard, *System 2* a desktop computer equipped with a simplified 17-key keyboard, *System 3* a tablet PC with an on-screen keyboard, *System 4* a tablet PC equipped with a simplified 17-key keyboard, *SD* standard deviation, *SRT* simple reaction time, *CRT* choice reaction time, *DA* digit addition, *SDS* symbol-digit substitution, *FTS* finger tapping speed, *RT*_*mean*_ mean reaction time, *SD*_*RT*_ standard deviation of reaction time, *Rate*_*CR*_ correct response rate, *FTS*_*D*_ finger tapping speed of dominant hand, *FTS*_*ND*_ finger tapping speed of non-dominant hand, *Group I* participants with typing speed of near zero (no competency using computers), *Group II* participants with typing speed less than 200 characters/min (relatively familiar with computers), *Group III* participants with typing speed of 200 characters/min or greater (very familiar with computers)

RT_mean_ and SD_RT_ are in millisecond (ms); FTS_D_ and FTS_ND_ are in average number of taps per 10 s

^*^Calculated by repeated measures ANOVA or Friedman test

^a^The numbers 1, 2, 3, and 4 represent Systems 1, 2, 3, and 4, respectively. Analyzed by Bonferroni or Wilcoxon signed-rank test

^b^Not statistically significant in post-hoc analysis

The mean reaction time for the SRT test showed a significant difference between systems for all three groups, and the post-hoc analyses demonstrated that the mean reaction time for the SRT test was greater for Systems 3 and 4 than for Systems 1 and 2 for all three groups, which was consistent with the results reported in Table 2. However, there were significant differences between Systems 1 and 2 for Group I and between Systems 3 and 4 for Group II. The mean reaction time for the CRT test also showed a significant difference between systems for all groups, and the post-hoc analyses showed the similar results to those reported in Table 2. In all groups, the mean reaction times for the CRT test using Systems 1 and 2 did not show significant differences and were greater than that for System 3. Performance using System 4, however, was superior to that using System 3 for Group II and Group III. The mean reaction time for the SDS test showed no statistically significant difference between systems for all three groups. Finally, performance on the DA and FTS tests did not differ by type of system, for all three groups.

## Discussion

### Overall performance between systems

Performance on the DA test did not differ significantly by test system. As for the FTS test, the performance using dominant hand did not demonstrate significant differences among test systems, but the test performed using non-dominant hand showed a significantly decreased performance in System 3 compared to Systems 1, 2 and 4. The DA test and the FTS_D_ did not seem to be influenced by the type of computers and input devices. On the other hand, for the SRT and CRT tests, and to some extent the SDS test, performance decreased on the tablet PC versus the desktop computer.

We postulated that participants who were unfamiliar with computers might show inferior performance because they would find it more difficult to adapt to the newly introduced tablet PC system. However, the performance of each computer familiarity group on the test systems showed similar patterns. For the SRT and CRT tests, all three computer familiarity groups uniformly showed decreased performance when tested with Systems 3 and 4. Moreover, reaction time for the SDS test also showed homogenous results among these groups. Therefore, computer unfamiliarity did not appear to lead to the decreased performance on the KCNT when using the tablet PC.

Accordingly, use of a tablet PC for the KCNT to evaluate performance on the SRT and CRT tests is not recommended; however, tablet PCs with an on-screen keyboard may be used to administer the DA test, and only limitedly the SDS and FTS tests.

### Systems 1 and 2: Full-key keyboard vs. simplified keyboard

The only difference between Systems 1 and 2 was the input method, that is, the keyboard. The results of this study showed that overall performance in four out of five subtests was slightly higher using a simplified keyboard than using a conventional one, but these differences in performance were not shown to be statistically significant for all subtests (Table 2).

Considering these results, there was no significant difference between the conventional full-key keyboard and the simplified keyboard in this study. Nevertheless, previous studies demonstrated that using a relatively complex conventional 106-key keyboard may lower examinees’ performance compared to using a simplified keyboard, and therefore, use of a simplified keyboard was recommended [9, 14].

### Systems 2 and 4: Bigger stimuli vs. smaller stimuli

Systems 2 and 4 were a desktop computer and a tablet PC, respectively, both equipped with a simplified keyboard. The difference between these two systems was the size of the display with other conditions kept equivalent. The performance results between Systems 2 and 4 suggested that the size of the display did not influence the tests related to higher cognitive functions (i.e., DA and SDS tests) but did influence the tests related to simple and basic cognitive functions (i.e., SRT and CRT tests). Similarly, previous studies also reported that diminished stimuli dimension led to a latency in reaction time [23]. Moreover, size, contrast, and luminance of visual stimuli have been shown to be major determinants of detection threshold affecting neurobehavioral performance on computerized tests [24].

On the other hand, Kim et al. used a simplified keyboard and found a laptop and desktop computer only showed marginal differences in performance, which were not statistically significant [15]. However, we believe that only minor differences were found because there was not much difference in the size of the display: the monitor had a display with a diagonal length of 17 in. and that of the laptop computer was 15 in. Kim’s study implies that, if the size of the display is similar, the platform of the KCNT system, whether a desktop or laptop computer, will not affect performance significantly.

Despite the aforementioned efforts to explain the results, it is not possible to claim with certainty that the size of stimuli was the only difference between System 2 and System 4 influencing the participants’ performance, because we have not compared a desktop computer against a tablet PC with similar screen sizes. However, it is certain that performance significantly differed when using the desktop computer versus tablet PC.

### Systems 3 and 4: On-screen keyboard vs. simplified keyboard

Systems 3 and 4 were based on a tablet PC with the same display size but different input devices. An on-screen keyboard was implemented in System 3 and a simplified keyboard was used in System 4. To our surprise, Systems 3 and 4 did not show any performance differences for almost all parameters except for the CRT and FTS tests, similar to how Systems 1 and 2 showed similar performances (Table 3). Although the difference in mean reaction time for the CRT test between Systems 3 and 4 was only 58.5 ± 81.2 ms, it was indeed statistically significant at *p* < 0.05. FTS_ND_ also showed a difference (3.2 ± 5.9 taps, *p* < 0.001), whereas FTS_D_ did not.

It seems that change of input method does not greatly influence the results of tests involving higher-order cognitive functions that require longer reaction times, such as the DA and SDS tests. Likewise, basic tasks such as the SRT and FTS_D_ tests barely require examinees to scan the keyboard because tapping a spacebar or a control key is all that is needed to complete the tests. Hence, no differences were observed between an on-screen keyboard and a simplified keyboard.

The CRT test, on the other hand, demands examinees to perceive stimuli on the display, scan arrow keys on the keyboard, and give correct responses as quickly as possible. Our interpretation of the results is that the simplified keyboard with tactile feedback was superior to the on-screen keyboard in such a test. While physical keyboards offer visual-auditory-tactile feedback, on-screen keyboards only provide visual-auditory feedback. The results of this study implied that the contribution of tactile feedback to the test performance was more substantial on non-dominant hand than dominant hand for the FTS test and on the CRT test than the SRT test. Numerous previous studies reported that tactile feedback improves performance of various tasks [25–28]. The fact that a conventional 106-key keyboard and a 17-key simplified keyboard provide the same type of feedback also explains why there was no significant difference for the CRT between Systems 1 and 2.

### Other considerations

#### Software and touchscreen latency

The KCN software used in this study was the KCN system by MaxMedica Inc. In its user’s guide, the minimum requirements for the system, such as the operating system, central processing unit, memory, disk space, and the display resolution, are clearly specified [29]. In this study, the desktop and tablet PC system both met these requirements. Therefore, it was reasonable to assume that they would produce results with the same level of accuracy. In addition, the maximum theoretical polling rate of a standard keyboard is 1000 Hz (i.e., every 1 ms), and the standard report rate of a capacitive screen digitizer installed on a mobile device is approximately 100 Hz (i.e., every 10 ms) [30]. However, the similar performance of the KCNT between Systems 3 and 4 implies that “touchscreen latency” was not a major contributor to consistently decreased performance on the tablet PC compared to that on the desktop computer. Given that the input device, whether the simplified keyboard or the on-screen keyboard, did not significantly influence the responsiveness of the participants, we carefully assume that the latency would have been reflected in the difference in the mean reaction time of the SRT test between Systems 3 and 4, which was at most 22.4 ms.

#### Standard deviation of the reaction time

The SD_RT_ for the CRT test was significantly larger when performed on the desktop computer than on the tablet PC (Tables 2 and 4), and it was also significantly larger for the SRT test on the desktop computer compared to the tablet PC in all three computer familiarity groups (Table 4). It suggests that the variability of the test results is greater on the tablet PC and also that the tests are less reliable than those performed on the desktop computer. Therefore, along with the decreased performance demonstrated in this study, it would not be recommended to build a test system with a tablet PC.

### Limitations

The relatively small number of participants in the computer familiarity groups is a limitation of this study. There were only 9 participants in Group I, whereas Groups II and III had 23 and 40 participants, respectively. This was because most participants were somewhat familiar with the use of computers. With the current high level of computer literacy in the population, obtaining a large number of participants unfamiliar with computers would require a much larger number of overall participants.

## Conclusions

This study evaluated and assessed performance on the KCNT in four different settings. It is not recommended to use a tablet PC for the KCNT to evaluate neurobehavioral performance for the SRT and CRT tests; however, tablet PCs with an on-screen keyboard may be used to perform the DA test, and only limitedly the SDS and FTS tests.

## Abbreviations

CRT: Choice reaction time
DA: Digit addition
DC: Digit classification
FTS: Finger tapping speed
FTS_D_: Average number of taps per 10 s using dominant hand
FTS_ND_: Average number of taps per 10 s using non-dominant hand
KCNT: Korean Computerized Neurobehavioral Test
Rate_CR_: Correct response rate
RT_mean_: Mean reaction time
SD_RT_: Standard deviation of reaction time
SDS: Symbol digit substitution
SRT: Simple reaction time
